# Effect of Folic Acid Supplementation on Cardiovascular Outcomes: A Systematic Review and Meta-Analysis

**DOI:** 10.1371/journal.pone.0025142

**Published:** 2011-09-28

**Authors:** Yu-Hao Zhou, Jian-Yuan Tang, Mei-Jing Wu, Jian Lu, Xin Wei, Ying-Yi Qin, Chao Wang, Jin-Fang Xu, Jia He

**Affiliations:** 1 Department of Health Statistics, Second Military Medical University, Shanghai, China; 2 Office of Compliance and Development, Center for Drug Evaluation, SFDA, Beijing, China; 3 Shanghai Jiao Tong University School of Medicine, Shanghai, China; University of British Columbia, Canada

## Abstract

**Background:**

Folic acid is widely used to lower homocysteine concentrations and prevent adverse cardiovascular outcomes. However, the effect of folic acid on cardiovascular events is not clear at the present time. We carried out a comprehensive systematic review and meta-analysis to assess the effects of folic acid supplementation on cardiovascular outcomes.

**Methodology and Principal Findings:**

We systematically searched Medline, EmBase, the Cochrane Central Register of Controlled Trials, reference lists of articles, and proceedings of major meetings for relevant literature. We included randomized placebo-controlled trials that reported on the effects of folic acid on cardiovascular events compared to placebo. Of 1594 identified studies, we included 16 trials reporting data on 44841 patients. These studies reported 8238 major cardiovascular events, 2001 strokes, 2917 myocardial infarctions, and 6314 deaths. Folic acid supplementation as compared to placebo had no effect on major cardiovascular events (RR, 0.98; 95% CI, 0.93–1.04), stroke (RR, 0.89; 95% CI,0.78–1.01), myocardial infarction (RR, 1.00; 95% CI, 0.93–1.07), or deaths from any cause (RR, 1.00;95% CI, 0.96–1.05). Moreover, folic acid as compared to placebo also had no effect on the following secondary outcomes: risk of revascularization (RR, 1.05; 95%CI, 0.95–1.16), acute coronary syndrome (RR, 1.06; 95%CI, 0.97–1.15), cancer (RR, 1.08; 95%CI, 0.98–1.21), vascular death (RR, 0.94; 95%CI,0.88–1.02), or non-vascular death (RR, 1.06; 95%CI, 0.97–1.15).

**Conclusion/Significance:**

Folic acid supplementation does not effect on the incidence of major cardiovascular events, stroke, myocardial infarction or all cause mortality.

## Introduction

Cardiovascular disease is the leading cause of premature morbidity and mortality worldwide for both men and women [1], [2]. Over the past few decades, many studies have shown a strong correlation between hyperhomocysteinemia and vascular disease [3]–[6], and identified elevated homocysteine levels as a risk factor for coronary artery disease, stroke, and deep vein thrombosis. Therefore, it has been suggested that raised concentrations of homocysteine in the blood should be lowered as a therapeutic approach to prevent cardiovascular disease [7], [8]. However, reduction of the concentrations of homocysteine in the blood has not consistently been shown to be beneficial [9]–[11].

Currently, folic acid and B vitamins are used for achieving target homocysteine levels, and are clearly effective at reducing concentrations of plasma homocysteine. However, their effects on vascular events remain unclear [12]. Additionally, several large-scale randomized controlled trials have shown that reducing the extent of homocysteinemia with folic acid does not improve cardiovascular outcomes. When combined with B vitamins, folic acid may actually accelerate the risk of cardiovascular disease, and this has further restricted its application in clinical prevention [13].

Recently, additional large-scale randomized controlled trials of folic acid therapy combined with other B vitamins have been completed [11], [14], [15]. A number of these trials indicated that combination therapy had some beneficial effect on cardiovascular events, whereas others showed that it had limited effects, and some even found that it could induce drug-related adverse reactions. This led to uncertainty over the presence and magnitude of any protective cardiovascular effects of folic acid and difficulties in interpretation of the results. For a better understanding of the effect of folic acid on homocysteine levels and cardiovascular outcomes, data from these recent trials need to be re-evaluated and combined with the data in former literature on folic acid. Therefore, we carried out a systematic review and meta-analysis of pooled data from randomized controlled trials to assess the possible effect of folic acid supplementation on major cardiovascular events.

## Methods

### Data sources, search strategy, and selection criteria

Randomized, double-blind, placebo-controlled, and trials of folic acid therapy in English-language literature were eligible for inclusion in our meta-analysis, regardless of publication status (published, unpublished, in press, and in progress), and the effects on homocysteine levels and cardiovascular outcomes were examined. Relevant trials were identified with the following procedure:

#### (1) Electronic searches

We searched the electronic databases Medline, EmBase, and the Cochrane Central Register of Controlled Trials for articles to a time limit of Nov. 20, 2010, using “folic acid”, “folate”, “cardiovascular disease”, “coronary disease”, “coronary thrombosis”, “ischemic heart disease”, “stroke”, “coronary stenosis”, “coronary restenosis”, and “randomized controlled trial” as the search terms. All reference lists from reports on non-randomized controlled trials were searched manually for additional eligible studies.

#### (2) Other sources

We contacted authors to obtain any possible additional published or unpublished data, and searched the proceedings of annual meetings in the Cochrane Cardiovascular Disease Group Specialized Register. In addition, we searched for ongoing randomized controlled trials, which had been registered as completed but not yet published, in the metaRegister of Controlled Trials. Medical subject headings and methods, patient population, and intervention were used to identify relevant trials. This review was conducted and reported according to the PRISMA (Preferred Reporting Items for Systematic Reviews and Meta-Analysis) Statement issued in 2009 (Checklist S1) [16].

The literature search was undertaken independently by 2 authors (Chao Wang and Ying-Yi. Qin) with a standardized approach, and any disagreement between these 2 authors was settled by a third author (Yu-Hao. Zhou) until a consensus was reached. All completed randomized controlled trials assessing the effects of folic acid therapy compared with the effects of a placebo, and reporting at least 1 outcome of major cardiovascular events were included as eligible trials. Randomized controlled trials to be included in the analysis were limited to those with at least 100 patients and at least 6 months follow-up, to ensure that only high-quality studies were incorporated.

### Data collection and quality assessment

Two investigators (Chun-Fang. Wu and Mei-Jing. Wu) independently extracted and collected data using a standardized data-extraction protocol. Any discrepancy was settled by group discussion, after which the primary authors (Yu-Hao. Zhou and Jia He) made the final decision. The data collected included baseline patient characteristics (number of patients, age, sex, pre-existing diseases, interventions, total plasma homocysteine, and duration of follow-up). The outcomes investigated included major cardiovascular events, stroke, myocardial infarction, total mortality, and possible drug-correlated adverse reactions. We measured the quality of the trials included in this study with the Jadad score [17] on the basis of randomization, concealment of treatment allocation, blinding, completeness of follow-up, and use of intention-to-treat analysis.

### Statistical analysis

We assessed the overall effect of folic acid supplementation on all data from the included trials. The outcomes were reported using relative risks (RR) with 95% confidence intervals (CIs) to estimate the effect of folic acid on major cardiovascular events, stroke, myocardial infarction, total mortality, and possible drug-correlated adverse reactions. After this, a subgroup analysis was carried out based on the number of patients, duration of folic acid supplementation, mean age, baseline total plasma homocysteine, pre-existing disease, and Jadad score. The statistical estimates of effect were derived using a random-effects model with Mantel–Haenszel statistics. Heterogeneity of treatment effects between studies was investigated visually by scatter plot analysis and statistically by the heterogeneity I^2^ statistic. I^2^ statistic of 0%–40% indicates unimportant heterogeneity, 30%–60% indicates moderate heterogeneity, 50%–90% indicates substantial heterogeneity, and 75%–100% indicates considerable heterogeneity [18]. P values were calculated by χ^2^ tests. All the reported P values are two-sided and value of P less than 0.05 was regarded as statistically significant for all included studies. All analyses were calculated using STATA (version 10.0).

## Results

**Figure 1:**
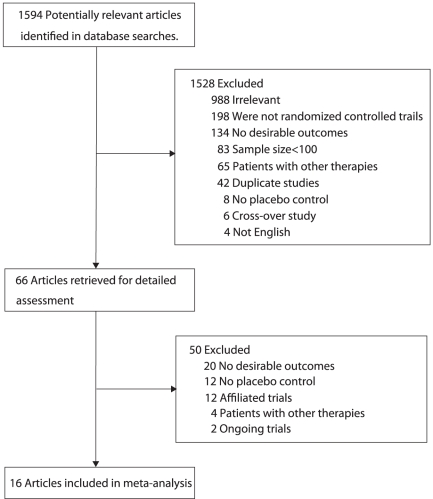
Flow diagram of the literature search and trial selection process.

We identified 1594 potentially relevant trials from our initial electronic search, and excluded 1528 trials after a preliminary review. The remaining 66 studies were retrieved for detailed assessment, and 16 randomized controlled trials met the inclusion criteria (Figure 1 and Protocol S1 [16]), which consisted of data of 44841 individual patients. Table 1 summarized the baseline characteristics of the participants and the design of the studies included. The trials included in this study compared folic acid supplementation (with or without B vitamins) with placebo. The follow-up for patients ranged from 8.3 to 87.6 months, with a mean of 43.2 months. The population of the trials ranged from 114 to 12064 individuals, with a mean of 2803. We restricted the inclusion criteria to randomized placebo-controlled trials with at least 100 patients and a minimum of 6 months follow-up to ensure that high-quality literature was included in our research, and to ensure a reliable conclusion. One trial had a Jadad score of 5, 6 trials had a score of 4, 7 trials had a score of 3, and the remaining 2 trials had a score of 2.

**Table 1:** Design and patient characteristics for trials included in the systematic review and meta-analysis.

Source	No. of patients	Mean age, y	Sex (male)	Pre-existent diseases	Intervention	Total homocysteine (µmol/L)	Duration of follow-up (months)	Jadad score
Andrew A.H [14] (2010)	238	60.4	178 (74.79%)	Diabetic nephropathy	2.5 mg folic acid	15.55	36	5
VITATOPS Study [15] (2010)	8164	62.6	5218 (63.91%)	Stroke	2 mg folic acid	14.3	40.8	4
Howard N.H [18] (2009)	506	61.4	309 (61.07%)	tHcy ≥8.5 µmol/L	5 mg folic acid	9.6	37.2	3
Marta E [19] (2008)	2319	61.7	1840 (79.34%)	CHD	0.8 mg folic acid	14.4	38	4
Christine M.A [20] (2008)	5442	62.8	0 (0%)	CHD	2.5 mg folic acid	NR	87.6	3
Jamison R.L [10] (2007)	2056	65.8	2023 (98.39%)	ACKD or ESRD	40 mg folic acid	22.4	38.4	3
Areuza V.A.V [21] (2007)	186	48.5	110 (59.14%)	ESKD	10 mg folic acid, 3 times a week	24.7	24	3
Bonaa K.H [22] (2006)	2815	63.0	2085 (74.07%)	CHD	0.8 mg folic acid	13.1	42	4
HOPE-2 Study [23] (2006)	5522	68.9	3963 (71.77%)	CHD	2.5 mg folic acid	12.2	60	4
ASFAST Study [24] (2006)	315	56.0	213 (67.62%)	CRF	15 mg folic acid	27.0	43.2	3
The Swiss Heart Study [25] (2002)	553	62.5	445 (80.47%)	Coronary stenosis	1 mg folic acid	NR	12	3
Mark S.D [26] (1995)	3318	54.0	1461 (44.03%)	Esophageal Dysplasia	0.8 mg folic acid	NR	72	2
Liem A [27] (2005)	593	65.2	462 (77.91%)	CHD	0.5 mg folic acid	12.1	42	2
SEARCH Collaborative Group [11] (2010)	12064	NR	10012 (82.99%)	MI	2 mg folic acid	13.5	80.4	4
Marco R [28] (2006)	114	64.4	63 (55.26%)	Hemodialysis	5 mg folic acid	31.7	29	4
Lange H [29] (2004)	636	61.3	490 (77.04%)	Coronary stenting	1 mg folic acid	12.6	8.3	3

**Figure 2:**
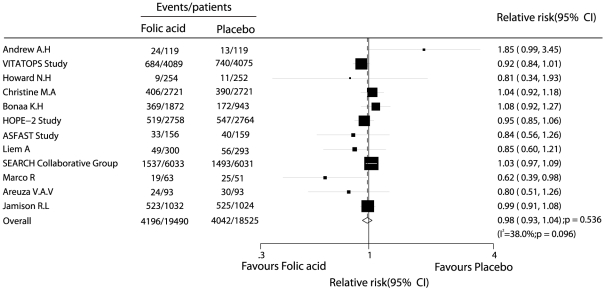
Effects of folic acid supplementation on the risk of major cardiovascular events.

Data for the effect of folic acid on major cardiovascular events were available from 12 trials, including 38015 individuals with 8238 cardiovascular events. Figure 2 shows the effect of folic acid (with or without B vitamins) on major cardiovascular events as compared to placebo. The pooled RR showed a 2% reduction in cardiovascular event rates, and with no evidence showed that folic acid therapy protected against cardiovascular event risk (RR, 0.98; 95%CI, 0.93–1.04). Although there was some evidence of heterogeneity across the studies included, a sensitivity analysis indicated that the results were not affected by sequential exclusion of any particular trial from all pooled analysis.

**Figure 3:**
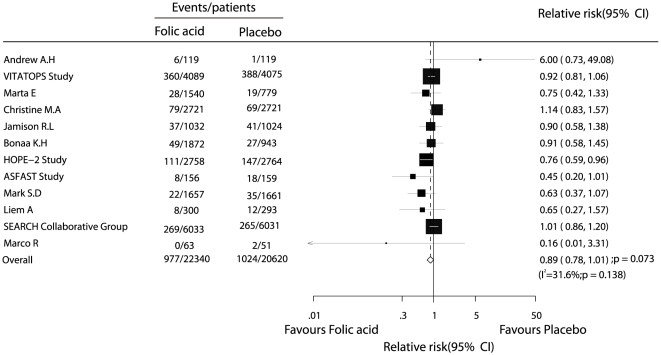
Effects of folic acid supplementation on the risk of stroke.

Data for the effect of folic acid on stroke were available from 12 trials, including 42960 participants with 2001 events of stroke. Overall, folic acid therapy reduced the risk of stroke by 11%, but was not associated with a statistically significant decrease in the risk of stroke (fatal or nonfatal) events (RR, 0.89; 95% CI, 0.78–1.01, with unimportant heterogeneity, Figure 3).

**Figure 4:**
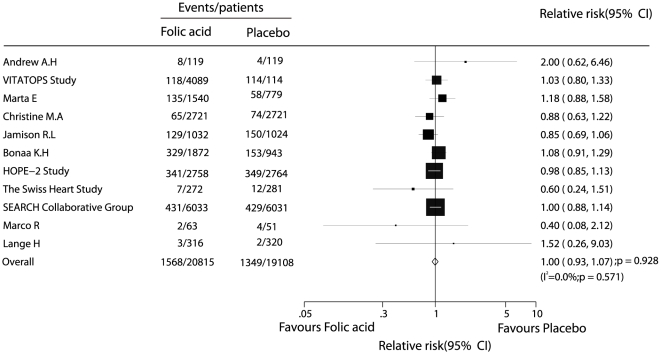
Effects of folic acid supplementation on the risk of myocardial infarction.

Data for the effect of folic acid on myocardial infarction were available from 11 trials, including 39923 patients and 2917 events of myocardial infarction. No effect of folic acid therapy on the risk of myocardial infarction events was observed (RR, 1.00; 95% CI, 0.93–1.07, without evidence of heterogeneity of effect, Figure 4).

**Figure 5:**
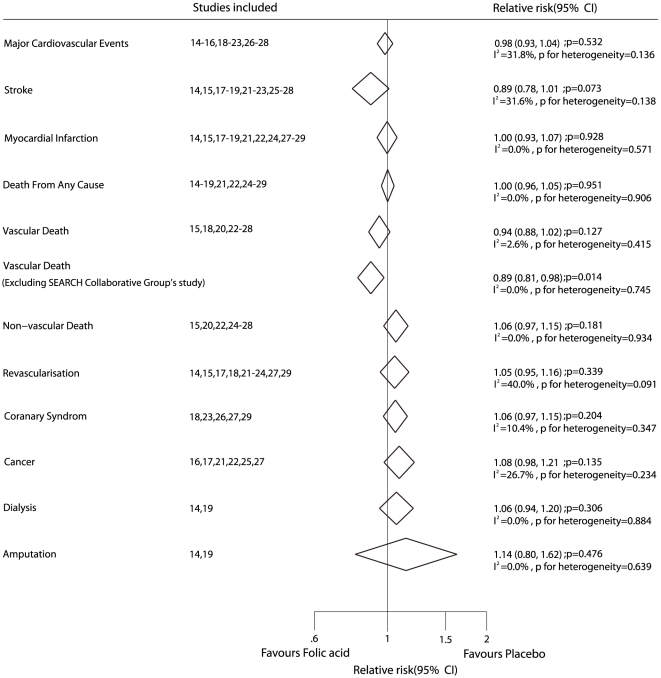
Summary of the relative risks of all outcomes assessed.

Fourteen trials including 44340 patients and 6314 total events of mortality were recorded, with 10 trials providing separate data for vascular death and 8 studies providing separate data for non-vascular death. There was no evidence to show that folic acid therapy could reduce the risk of mortality, whether total mortality, vascular death, or non-vascular death (Figure 5). According to a sensitivity analysis, we excluded the (SEARCH) Collaborative Group study [11]. This trial specifically included individuals with pre-existing myocardial infarction, which may have contributed to a high mortality rate. After this, we could conclude that folic acid therapy was associated with a reduction in the risk of vascular death, which was decreased by 11% (RR, 0.89; 95% CI, 0.81–0.98, Figure 5).

Ten of the trials included 38068 patients with 2939 revascularization events. There was no evidence to show that folic acid therapy protected against revascularization, although heterogeneity was observed in the magnitude of the effect across the trials included (RR, 1.05; 95% CI, 0.95–1.16, Figure 5). However, after sequential exclusion of each trial from all pooled analysis, the results were not affected by exclusion of any specific trial.

The risk of coronary syndrome was reported in 5 trials, including 19050 individuals and 3148 events of coronary syndrome. No evidence indicated that folic acid therapy protected against coronary syndrome risk (RR, 1.06; 95%CI, 0.97–1.15, without evidence of heterogeneity of effect, Figure 5).

Six trials reported data for the incidence of cancer, including 26544 patients and 2472 events of cancer. Reduction in the risk of cancer with folic acid therapy was not statistically significant (RR, 1.08; 95%CI, 0.98–1.21, with unimportant heterogeneity, Figure 5).

Of the 16 trials included in our meta-analysis, only 2 provided data about dialysis and amputation, and included 2294 participants, 725 dialysis events, and 116 amputation events. The pooled analysis showed no significant differences between folic acid therapy and placebo therapy for dialysis or amputation (Figure 5).

**Figure 6:**
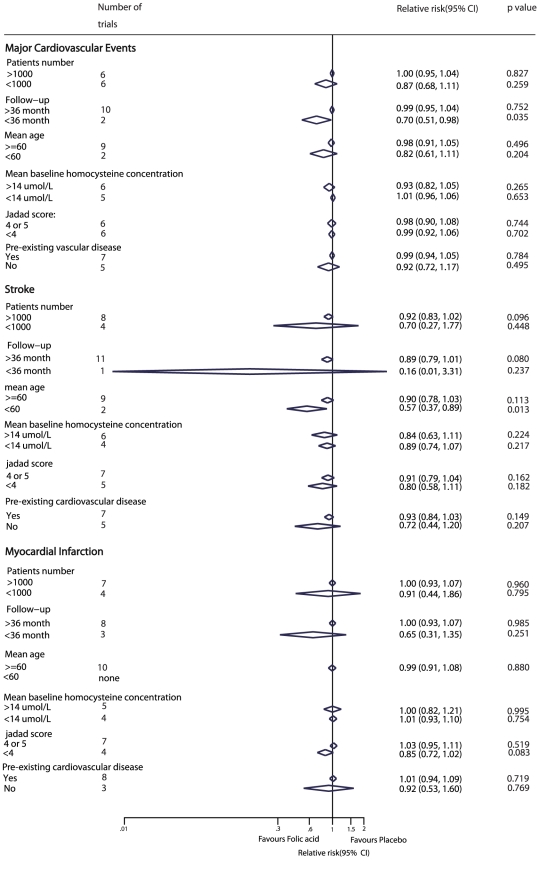
Subgroup analysis for the effect of folic acid supplementation on major cardiovascular events, stroke, and myocardial infarction.

Subgroup analyses were carried out for major cardiovascular events, stroke, and myocardial infarction. We noted that folic acid therapy was associated with a reduction in the risk of major cardiovascular events, when trials with less than 36 months follow-up period were included. Furthermore, compared with placebo, folic acid therapy showed a clear effect on stroke events when the mean age of the patients was less than 60 years. However, no other significant differences were identified between the effect of folic acid therapy and placebo, based on additional subset factors (Figure 6).

## Discussion

This large quantitative review included 44841 individuals in 16 trials with a broad range of baseline characteristics. The results of our meta-analysis suggest that folic acid therapy does not effect on the incidence of major cardiovascular events, stroke, myocardial infarction, all cause mortality or other cardiovascular-related outcomes.

The relationship between homocysteine levels and cardiovascular disease was described initially by observational studies, which may overestimate the effect of this relationship. Two meta-analyses of epidemiologic studies [4], [31] suggested that reduced homocysteine levels could lower the risk of coronary heart disease, stroke, and cardiovascular disease. However, Bazzano et al [32] concluded that folic acid therapy did not significantly contribute to cardiovascular disease, stroke, or myocardial infarction. Therefore, we carried out a systematic review and meta-analysis to explain the possible effect of folic acid on major cardiovascular events, and possible drug-correlated adverse reactions. This study was based on randomized controlled trials and explored any possible correlation between folic acid supplementation and the outcomes of any cardiovascular-related disease.

Our main findings are in contrast with the findings of previous epidemiologic research [4], [31], and also support the conclusion made by Bazzano et al [32] that folic acid had no significant benefit or adverse effect on the risk of major cardiovascular events, stroke, myocardial infarction, or any other related disease.

No significant differences in the relative risk of cardiovascular disease were reported across a wide background of cardiovascular risk in these trials. In our meta-analysis, participants with a history of cardiovascular disease or stroke [11], [15], [20], [21], [23], [24], [28], end-stage renal disease [10], [22], [29], chronic renal failure [25], diabetic nephropathy [14], coronary stenting [26], [30], esophageal dysplasia [27], and hyperhomocysteinemia [19] were included. However, an unimportant heterogeneity was reported for the included trials. Another important factor that may have affected the results is the plasma homocysteine levels. Subgroup analysis (Figure 6), based on baseline plasma homocysteine levels, was used to explore any possible variations. The reason for the absence of an effect of folic acid could be that the extent of the reduction in homocysteine was not reported in many trials, thus we were unable to assess the correlation between the level of reduction in homocysteine and cardiovascular outcomes.

There were no significant differences between folic acid supplementation and placebo in the relative risk for major cardiovascular events, myocardial infarction, or stroke. The reason for this could be that although elevated homocysteine levels impair vascular function, the impact of homocysteine on vascular outcomes is not primarily related to the pathogenesis of coronary artery disease [33], [34], which is largely attributable to plaque formation and rupture. Furthermore, high doses of B_6_ vitamins may adversely affect vascular remodeling and myocardial repair, and may therefore play an important role in increasing the rates of complications and death among patients with cardiovascular risk factors or pre-existing history of cardiovascular events [34], [35]. Therefore, although folic acid may have direct beneficial effects on cardiovascular outcomes in patients, these effects may be reduced or balanced by the adverse effects of high doses.

Folic acid supplementation may play an important role in carcinogenesis, because when it is administered to individuals with established cancers, it potentially promotes tumor growth [36], [37]. It has also been reported that the introduction of folic acid may increase the risk of colorectal cancer [38]. According to our review, folic acid therapy resulted in an 8% increase in the risk of cancer, although this difference was not statistically significant. The reason for this increase in carcinogenesis can be explained by the fact that folic acid supplementation may affect endothelial function and support cell growth through mechanisms independent of homocysteine [39].

Importantly, folic acid and B vitamins are water-soluble and excreted by the kidney; therefore, therapy toxicity may be of great concern in patients with impaired renal function. In patients with end-stage renal failure who have hyperhomocysteinemia wherein homocysteine levels must be reduced, alternative, non-vitamin therapies are important. For example, enhancing urinary excretion can help to avoid a decrease in glomerular filtration rate and an increase in major cardiovascular events [35], [40].

A previous meta-analysis [32] has illustrated that the risk of cardiovascular outcomes is not significantly reduced using folic acid supplementation compared with placebo; this conclusion was similar to our current meta-analysis. In our research, subgroup analysis suggested that folic acid supplementation contributes to a causal relationship with the risk of major cardiovascular events and stroke; however, these conclusions may be unreliable, because a smaller number of trials were included in such subsets. In addition, we did not include small trials with less than 100 patients and 6 months follow-up to ensure that the quality of studies included were comparable to that of a study by Bazzano et al [32]. Finally, the initial evidence for a correlation between folic acid supplementation and cardiovascular disease was provided by observational studies, which may overestimate the size of the effect. Our meta-analysis was restricted to randomized controlled trials to meet our inclusion criteria and aimed to provide the best evidence for a causal relationship.

The limitations of our research are as follows: (i) The extent of homocysteine lowering was unclear owing to the lack of data, and therefore, we were unable to explore the association between the levels of homocysteine and cardiovascular events. (ii) Although subgroup analysis suggested that folic acid supplementation significantly reduced the risk of major cardiovascular events for patients with follow-up of less than 36 months or decreased the risk of stroke for those patients with a mean age of less than 60 years, these results may be variable because of the small number of trials that were included in such subsets. (iii) Inherent assumptions made for any meta-analysis, because the analysis used pooled data either published or provided by individual study authors, and individual patient data or original data were unavailable, which restricted us doing more detailed relevant analysis and obtaining more comprehensive results.

The findings of this study suggested that folic acid supplementation had no significant effects on major cardiovascular events, stroke, myocardial infarction or all cause mortality . Furthermore, high-doses folic acid supplementation may increase the risk of cancer cell growth [36] and impair renal function [35]. Therefore, in future research, it is important to focus on healthy individuals for primary prevention of cardiovascular disease, and to combine other homocysteine-lowering drugs to provide an optimal therapy that minimizes adverse effects in patients with hyperhomocysteinemia. We suggest that the ongoing trials be improved in the following ways: (i) The adverse effects in clinical trials should be recorded and reported normatively, so that the side-effects of any treatment can be evaluated in future trials. ii) The role of treatment duration and dosage should be investigated in more detail to explore optimal dose and duration of treatment.

## Supporting Information

Checklist S1 **PRISMA Checklist.**
(DOC)

Protocol S1 **PRISMA Flowchart.**
(DOC)
