# Supplementary material for: Effect of Folic Acid Supplementation on Cardiovascular Outcomes: A Systematic Review and Meta-Analysis
Source: PLoS One. 2011 Sep 28;6(9):e25142. doi: 10.1371/journal.pone.0025142 (PMC3182189; doi:10.1371/journal.pone.0025142)
Supplement: Protocol S1 — PRISMA Flowchart. (DOC) [file pone.0025142.s002.doc]

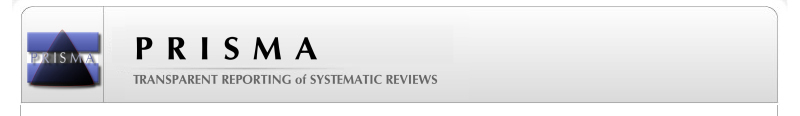
**PRISMA 2009 Flow Diagram**

**Screening**

**Included**

**Eligibility**

**Identification**

1594 Potentially relevant articles in database searches

1528 Excluded

988 Not relevant

198 Not RCTs

134 No desirable outcomes

83 Sample size<100

65 Patients with other therapies

42 Duplicate studies

8 No placebo control

6 Cross-over study

4 Not English

66 Articles retrieved for detailed assessment

50 Excluded

20 No desirable outcomes

12 No placebo control

12 Affiliated trials

4 Patients with other therapies

2 Ongoing trials

16 Articles included in meta-analysis
